# Incidence and Risk Factors of Delayed Chemotherapy‐Induced Nausea and Vomiting Among Adult Patients: A Cross‐Sectional Study

**DOI:** 10.1002/nop2.70490

**Published:** 2026-04-03

**Authors:** Mimi Zheng, Qi Zhang, Liping Ni, Zheng Zheng, Ying Wang, Yiwen Chen, Jing Huang, Jian Gao, Quanlei Li, Yuxia Zhang

**Affiliations:** ^1^ Nursing Department Zhongshan Hospital, Fudan University Shanghai People's Republic of China; ^2^ Nutrition Department Zhongshan Hospital, Fudan University Shanghai People's Republic of China; ^3^ School of Nursing, LKS Faculty of Medicine The University of Hong Kong Hong Kong People's Republic of China

**Keywords:** cancer, chemotherapy, delayed nausea and vomiting, influencing factor, nursing, patient reported outcome measures

## Abstract

**Aim:**

To assess the incidence of delayed chemotherapy‐induced nausea and vomiting (CINV) and identify key risk factors among adult patients.

**Design:**

A descriptive cross‐sectional design.

**Methods:**

A total of 326 adult patients undergoing chemotherapy were recruited using consecutive sampling from a nearly 4000‐bed tertiary general hospital in Shanghai, China, between July 2023 and April 2024. Demographic and health status data were collected within 24 h of patient admission. Clinicopathologic information was retrieved from nursing documents and electronic medical records. Delayed CINV was measured on the third day post‐chemotherapy via telephone interviews using the Common Terminology Criteria for Adverse Events (CTCAE 5.0). Statistical analyses included univariate analysis, random forest modelling, least absolute shrinkage and selection operator (LASSO) analysis and binary logistic regression.

**Results:**

Among 326 participants, 113 (34.7%) reported delayed nausea and/or vomiting, with 32.2% (*n* = 105) experiencing delayed nausea and 13.2% (*n* = 43) experiencing delayed vomiting. The step‐by‐step analysis identified anxiety, motion sickness and high emetogenicity of chemotherapy agents as key risk factors for delayed CINV. Post hoc analysis showed that body mass index (BMI) and history of delayed nausea or vomiting during previous chemotherapy cycles emerged as important predictors that should be considered when assessing the risk of delayed CINV.

**Conclusions:**

Health care providers should be vigilant in recognising patients at high risk for delayed CINV. Early and targeted prophylactic interventions are recommended to mitigate the risk of delayed CINV.

**Reporting Method:**

The study followed the STROBE checklist.

**Patient or Public Contribution:**

No patient or public contribution.

## 1 Introduction

Globally, cancer is one of the leading causes of morbidity and mortality, with an estimated 19.3 million new cases and almost 10 million deaths in 2020 (Sung et al. 2021). Cancer still presents a significant public health challenge for both high‐income countries and low‐ and middle‐income countries. China is the second largest economy with huge disparities and remains one of the most populous countries, with a population of 1.4 billion people; accordingly, it has the highest proportion of new cancer cases and deaths worldwide. According to the latest national report, over 4.8 million new cases and 2.6 million deaths were estimated to occur in China in 2022, making it the leading cause of death in the country (Han et al. 2024). Chemotherapy is one of the most commonly used treatments for cancer and has significantly improved survival rates. However, chemotherapy is often accompanied by severe side effects, among which chemotherapy‐induced nausea and vomiting (CINV) is one of the most distressing (Gupta et al. 2021).

CINV is typically categorised into two phases: acute and delayed. Acute CINV occurs within the first 24 h post‐chemotherapy treatment, when serotonin release plays a dominant role, and antiemetics are often more effective (Wickham 2012). In contrast, delayed CINV, which manifests 24 to 72 h post‐treatment, is driven by different neurotransmitters and involves multiple neurotransmitter pathways, making it more difficult to control (Wickham 2012). Consequently, delayed CINV often has a greater impact on patients' quality of life due to its prolonged nature and less effective management. The incidence of delayed CINV varies greatly among patients, with delayed nausea occurring in 49%–75% of cases and delayed vomiting in 19%–33%, depending on multiple factors such as the emetogenic risk of chemotherapy regimen, a prior history of nausea or vomiting, patient anxiety and the use of antiemetic prophylaxis (Gupta et al. 2021; Wickham 2012). Severe cases of delayed CINV can result in significant complications, including dehydration, electrolyte imbalances, malnutrition and may lead to delays or discontinuation of essential cancer treatments. In addition, the economic impact of delayed CINV is also considerable, as it often requires extra hospital visits, medical interventions and increased financial expenditure (Schwartzberg et al. 2015). A retrospective cohort study in the United States involving 19,139 patients found that 13.7% had hospital visits for delayed CINV, compared with only 0.2% for acute CINV, with a mean cost of CINV‐related hospital visit of $599 (Burke et al. 2011).

A significant challenge in managing delayed CINV is its occurrence outside the hospital setting, where health care providers may not readily observe it, and patients may also fail to report their symptoms. Studies have highlighted a disparity between health care providers' estimates of CINV incidence, with over 75% of delayed CINV cases being underestimated (Grunberg et al. 2004). This underestimation underscores the need for better preventive strategies to manage delayed CINV effectively. Given the high incidence of delayed CINV, its multifaceted negative consequences and the frequent underestimation by health care providers, it is imperative to develop targeted preventive strategies for effective management of delayed CINV in clinical practice. Therefore, in this study, we aimed to assess the incidence of delayed CINV among patients undergoing chemotherapy and to identify potential risk factors.

## 2 Background

CINV has been recognised as one of the most debilitating side effects of cancer treatment, and the impact of CINV on patients cannot be overstated (Gupta et al. 2021). Severe nausea and vomiting can lead to significant physical and psychological distress, interfere with daily activities and reduce the overall quality of life. Moreover, delayed CINV can result in treatment delays, compelling up to 20% of patients to postpone or even refuse future treatment (Jordan et al. 2007). Historically, CINV was considered an unavoidable consequence of chemotherapy, but advancements in antiemetic therapy have significantly reduced its incidence, particularly in the acute phase. However, delayed CINV, which occurs more than 24 h after chemotherapy, has been less studied than its acute counterpart.

The management of delayed CINV remains suboptimal, and delayed CINV continues to be a significant clinical challenge (Yuan and Committee of Neoplastic Supportive‐Care 2023). This is largely due to the complex pathophysiology of CINV, which involves multiple neurotransmitter pathways. Chemotherapeutic agents have been considered to trigger vomiting by activating neurotransmitter receptors located in the gastrointestinal tract, the vomiting centre and the chemoreceptor trigger zone in the brain (Gonella and Di Giulio 2015). It is believed that the mechanisms underlying delayed CINV differ from those of acute CINV, with the neurokinin‐1 (NK‐1) receptor playing a crucial role (Yuan and Committee of Neoplastic Supportive‐Care 2023). The delayed phase is less responsive to traditional antiemetic regimens, which primarily target serotonin receptors. The NK‐1 receptor, in particular, has been identified as a critical mediator in delayed CINV. This has led to the development of NK‐1 receptor antagonists (NK‐1RA), which have been shown to be more effective in managing delayed CINV (Hesketh 2001).

A systematic review of 49 studies identified 28 individual risk factors that significantly influence the risk of CINV (Mosa et al. 2020). Among these, at least five studies consistently highlighted seven key risk factors: a history of nausea/vomiting, female sex, expectation of CINV, younger age, anxiety, history of morning sickness and low alcohol intake. Despite these findings, it remains uncertain whether these factors are equally predictive of the incidence of delayed CINV. Furthermore, there remains considerable variability in the reported incidence of delayed CINV, suggesting that other, yet unidentified factors may also play a role.

## 3 Methods

### 3.1 Design

This study used a descriptive cross‐sectional design. The study was reported following the STROBE checklist for cross‐sectional studies (von Elm et al. 2007).

### 3.2 Setting and Participants

The study was conducted at a nearly 4000‐bed tertiary general hospital in Shanghai, China, which serves as a university‐affiliated teaching and research institution. The hospital employs over 2000 physicians and 2500 nurses and is one of the largest health care institutions in East China. It provides both general and specialised medical services to patients from local, regional and national levels. Annually, the hospital manages approximately 5.5 million emergency and outpatient visits, along with around 200,000 hospital admissions and 160,000 surgical procedures.

Participants in this study were adult patients undergoing chemotherapy. Inclusion criteria were: (a) patients aged 18 years and above; (b) those who had completed at least one cycle of chemotherapy via intravenous injection; (c) individuals capable of providing written informed consent and willing to participate voluntarily. Exclusion criteria were: (a) known organic conditions directly causing nausea or vomiting, such as gastrointestinal obstruction or intracranial hypertension; (b) factors that would confound CINV assessment, including nausea or vomiting within 24 h before the index chemotherapy or concurrent participation in another clinical trial; (c) severe cognitive or psychiatric impairment precluding effective communication; (d) sudden clinical deterioration as determined by the physician. Patients were excluded if they experienced a sudden aggravation of their disease. To minimise selection bias, consecutive sampling was used to ensure that all eligible participants were systematically included during the recruitment period.

The sample size was calculated using G*Power 3.1 to ensure adequate power to detect differences in the proportions of delayed CINV across demographic and clinicopathologic variables using a chi‐square test (Kang 2021). Assuming a medium effect size (*w* = 0.30), a two‐sided alpha level of 0.05 and power of 0.95, the minimum required sample size was 191. A separate sample size calculation was performed for the planned binary logistic regression analysis. Assuming an odds ratio of 1.50, with the same two‐sided alpha level (*α* = 0.05) and power (0.95), the minimum required sample size was 280. The final enrolled sample (*n* = 326) exceeded the most conservative requirement.

### 3.3 Instruments

#### 3.3.1 General Information Questionnaire

A self‐designed general information questionnaire was employed to gather demographic, health status and clinicopathologic data. In addition to demographic data such as age and gender, this study investigated variables related to health status (*n* = 6) and clinicopathologic information (*n* = 4) as potential risk factors for delayed CINV. These variables were selected based on a comprehensive literature review, which identified commonly reported predictors of delayed CINV (Mosa et al. 2020).

The demographic and health status variables included age, gender, body mass index (BMI), sporadic alcohol use, eating before starting chemotherapy, sleep disorders, motion sickness and anxiety. BMI was calculated as weight in kilograms divided by height in metres squared. Sporadic alcohol use was defined as occasional or irregular alcohol consumption prior to chemotherapy. Eating before starting chemotherapy referred to food intake just prior to receiving treatment. Sleep disorders were identified by the presence of symptoms such as difficulty falling or staying asleep. Motion sickness was assessed based on self‐reported previous experiences. Anxiety was measured by asking about feelings of anxiety since starting chemotherapy. All these variables including sporadic alcohol use, eating before starting chemotherapy, sleep disorders, motion sickness and anxiety were recorded as binary responses (yes or no), indicating the presence or absence of each condition.

Clinicopathologic variables encompassed the number of chemotherapy cycles completed, number of antiemetics used in the current cycle, history of delayed nausea or vomiting during previous chemotherapy cycles and high emetogenicity of chemotherapy agents. The history of delayed nausea or vomiting during previous chemotherapy cycles was recorded as a binary variable (yes or no). The emetogenicity of chemotherapy agents was classified according to the American Society of Clinical Oncology (ASCO) guidelines (Hesketh et al. 2020), which categorise agents as minimal, low, moderate or high. For the purpose of data analysis, this variable was recoded into a binary format (yes or no), differentiating between high emetogenicity and non‐high emetogenicity (which included minimal, low and moderate levels).

#### 3.3.2 The Common Terminology Criteria for Adverse Events

The severity of delayed CINV was assessed using the Common Terminology Criteria for Adverse Events (CTCAE) version 5.0. This scale classifies nausea into four grades and vomiting into five grades. Chemotherapy‐induced nausea was evaluated on a 4‐point Likert Scale: Grade 0 (no symptoms), Grade 1 (decreased appetite without altered eating habits), Grade 2 (reduced oral intake without significant weight loss, dehydration or malnutrition) and Grade 3 (inadequate oral intake requiring nasal feeding, total parenteral nutrition or hospitalisation). Chemotherapy‐induced vomiting was assessed on a 5‐point Likert scale: Grade 0 (no symptoms), Grade 1 (no intervention required), Grade 2 (outpatient intravenous rehydration or medical intervention required), Grade 3 (requires nasal feeding, total parenteral nutrition or hospitalisation), Grade 4 (life‐threatening) and Grade 5 (death). The CTCAE is widely used globally for assessing adverse effects in patients undergoing chemotherapy and has demonstrated satisfactory psychometric properties (Freites‐Martinez et al. 2021).

### 3.4 Data Collection

Data were collected between July 2023 and April 2024 from the medical oncology and haematology departments at the study hospital. The research was supported by logistical assistance and facilitation from the head nurses in each department. Two master‐prepared research assistants, who were practicing nurses in the respective departments, were appointed and trained by the research team members (M.Z. and Q.Z.). The training standardised data collection procedures, including participant recruitment, informed consent, record extraction and telephone interviews. The quality of data collected from the initial 10 patients was meticulously reviewed by the research team, and no significant issues were identified. Additionally, a random selection of three patient cases was examined each month for quality assurance throughout the study until data collection was completed.

Demographic and health status data were collected via face‐to‐face interviews within 24 h of patient admission, while clinicopathologic information was retrieved from nursing documents and electronic medical records when available. The severity of delayed CINV was assessed by research assistants on the third day following the completion of the current chemotherapy cycle through telephone interviews. During data collection, any instances of missing data were minimised by closely monitoring data entry and promptly following up with participants to ensure complete and accurate responses. To mitigate recall bias, all interviews were conducted within a consistent timeframe post‐admission and post‐chemotherapy, and self‐reported data were cross‐verified with medical records to ensure accuracy and reliability. Additionally, measures were implemented to reduce social desirability bias by assuring participants that their responses would remain confidential and would not influence their care. Research assistants were trained to use neutral, non‐judgmental language during interviews to further minimise the likelihood of participants providing socially desirable responses.

### 3.5 Statistical Analysis

Data were presented as median and interquartile range (IQR) for continuous variables and as counts and proportions for categorical variables. Differences in the incidence of delayed CINV across demographic, health status and clinicopathologic variables were analysed using Mann‐Whitney *U* test and chi‐square test, as appropriate. A random forest model was constructed using the R package RandomForest to assess and rank the importance of variables, followed by variable filtering through least absolute shrinkage and selection operator (LASSO) analysis using the glmnet function. The importance of each variable was assessed using the percentage increase in mean squared error (%IncMSE), where a higher %IncMSE value indicates a greater influence of the variable on the model's prediction accuracy. A LASSO analysis was conducted to refine the list of significant variables, with the optimal lambda (*λ*) value identified during the process. The optimal lambda value represents the point at which the model achieves the best balance between bias and variance, minimising prediction error. This value controls the strength of the regularisation applied by LASSO, effectively shrinking some regression coefficients to zero and thereby excluding non‐essential variables from the model. This technique is particularly effective for both variable selection and regularisation, enhancing the model's predictive accuracy by reducing overfitting and improving generalizability (Musoro et al. 2014). A binary logistic regression analysis was then performed to determine the strength and direction of the relationships between the filtered variables and delayed CINV (Ng et al. 2023). All statistical tests were two‐sided, with the level of significance established at *p* < 0.05. Statistical analyses were conducted using SPSS Version 26 and R Studio software (2024.04.0).

### 3.6 Ethical Considerations

This study was approved by the Medical Ethics Committee of Zhongshan Hospital, Fudan University (Reference number: B2024‐098R). Written informed consent was obtained from all eligible participants prior to data collection. Participants were informed of their right to withdraw from the study at any point without penalty. Participants were also provided with the contact information of the research assistants in case they had any inquiries regarding the study. No financial incentives were offered to participants.

## 4 Results

### 4.1 Participants Characteristics

A total of 326 eligible patients undergoing chemotherapy were included in this analysis with a response rate of approximately 90%. The median age of the participants was 60 years (52–69), with over half being male (*n* = 193, 59.2%). The median BMI was 23.1 (21.1–25.6).

Sporadic alcohol use was reported by the majority of participants (*n* = 238, 73.0%). Similarly, 76.4% (*n* = 249) of the participants reported eating before starting chemotherapy. Fewer participants reported sleep disorders (*n* = 25, 7.7%), motion sickness (*n* = 18, 5.5%) or anxiety (*n* = 16, 4.9%).

The median number of chemotherapy cycles completed and the median number of antiemetics used in the current cycle were 3 (2–5) and 2 (1–3), respectively. Additionally, 86 participants (26.4%) reported a history of delayed nausea or vomiting during previous chemotherapy cycles. Chemotherapy agents with high emetogenicity were used in 99 participants (30.4%).

### 4.2 Incidence of Delayed CINV

Among the 326 participants, 113 (34.7%) reported delayed nausea and/or vomiting. More specifically, 105 (32.2%) experienced delayed nausea, with the majority (*n* = 95, 90.5%) classified as Grade 1, and the remaining 10 participants (9.5%) experiencing Grade 2 delayed nausea. Additionally, 43 participants (13.2%) developed delayed vomiting, all of which were classified as Grade 1.

### 4.3 Identification and Filtering of Factors Associated With Delayed CINV

#### 4.3.1 Univariate Analysis

Table 1 shows the univariate analysis of various potential risk factors for delayed CINV. Gender, BMI, sporadic alcohol use, motion sickness, anxiety and high emetogenicity of chemotherapy agents were associated with the risk of delayed nausea. Similarly, BMI, sporadic alcohol use, anxiety, history of delayed nausea or vomiting and high emetogenicity of chemotherapy agents were associated with the risk of delayed vomiting.

**TABLE 1:** Univariate analysis of risk factors for delayed CINV (*N* = 326).

Variable	Total (*N* = 326)	Delayed nausea				Delayed vomiting			*p*
		With delayed nausea (*n* = 105)	Without delayed nausea (*n* = 221)	Statistics	*p*	With delayed vomiting (*n* = 43)	Without delayed vomiting (*n* = 283)	Statistics	
Age, year	60 (52–69)	59 (47.5–69.5)	60 (53.5–69)	−1.622^a^	0.105	53 (43–72)	60 (53–69)	−1.096^a^	0.273
Gender									
Male	193 (59.2%)	49 (46.7%)	144 (65.2%)	10.077^b^	0.002**	20 (46.5%)	173 (61.1%)	3.303^b^	0.069
Female	133 (40.8%)	56 (53.3%)	77 (34.8%)			23 (53.5%)	110 (38.9%)		
BMI	23.1 (21.1–25.6)	22.2 (20.2–23.8)	23.9 (21.4–26.0)	−4.021^a^	< 0.001**	21.4 (20.1–23.4)	23.3 (21.3–25.8)	−3.110^a^	0.002**
Sporadic alcohol use									
Yes	238 (73.0%)	88 (83.8%)	150 (67.9%)	9.173^b^	0.002**	37 (86.0%)	201 (71.0%)	4.274^b^	0.039*
No	88 (27.0%)	17 (16.2%)	71 (32.1%)			6 (14.0%)	82 (29.0%)		
Eating before starting chemotherapy									
Yes	249 (76.4%)	77 (73.3%)	172 (77.8%)	0.797^b^	0.372	37 (86.0%)	212 (74.9%)	2.565^b^	0.109
No	77 (23.6%)	28 (26.7%)	49 (22.2%)			6 (14.0%)	71 (25.1%)		
Sleep disorders									
Yes	25 (7.7%)	9 (8.6%)	16 (7.2%)	0.178^b^	0.673	1 (2.3%)	24 (8.5%)	1.223^b^	0.269
No	301 (92.3%)	96 (91.4%)	205 (92.8%)			42 (97.7%)	259 (91.5%)		
Motion sickness									
Yes	18 (5.5%)	15 (14.3%)	3 (1.4%)	22.806^b^	< 0.001**	5 (11.6%)	13 (4.6%)	2.321^b^	0.128
No	308 (94.5%)	90 (85.7%)	218 (98.6%)			38 (88.4%)	270 (95.4%)		
Anxiety									
Yes	16 (4.9%)	15 (14.3%)	1 (0.5%)	29.185^b^	< 0.001**	8 (18.6%)	8 (2.8%)	16.673^b^	< 0.001**
No	310 (95.1%)	90 (85.7%)	220 (99.5%)			35 (81.4%)	275 (97.2%)		
Number of chemotherapy cycles completed	3 (2–5)	3 (2–5)	3 (2–5)	−0.022^a^	0.983	2 (1–5)	3 (2–5)	−1.555^a^	0.120
Number of antiemetics	2 (1–3)	2 (1–3)	2 (1–3)	−0.483^a^	0.629	2 (1–3)	2 (1–3)	−0.819^a^	0.413
History of delayed nausea or vomiting									
Yes	86 (26.4%)	35 (33.3%)	51 (23.1%)	3.855^b^	0.050	22 (51.2%)	64 (22.6%)	15.664^b^	< 0.001**
No	240 (73.6%)	70 (66.7%)	170 (76.9%)			21 (48.8%)	219 (77.4%)		
High emetogenicity									
Yes	99 (30.4%)	47 (44.8%)	52 (23.5%)	15.175^b^	< 0.001**	21 (48.8%)	78 (27.6%)	7.990^b^	0.005**
No	227 (69.6%)	58 (55.2%)	169 (76.5%)			22 (51.2%)	205 (72.4%)		

*Note:* Data are presented as *n* (%) or median (interquartile range). *p < 0.05, **p < 0.01.

Abbreviation: BMI, body mass index.

^a^ Z score.

^b^ Chi‐square value.

#### 4.3.2 Ranking of Variables Based on Importance

**FIGURE 1:**
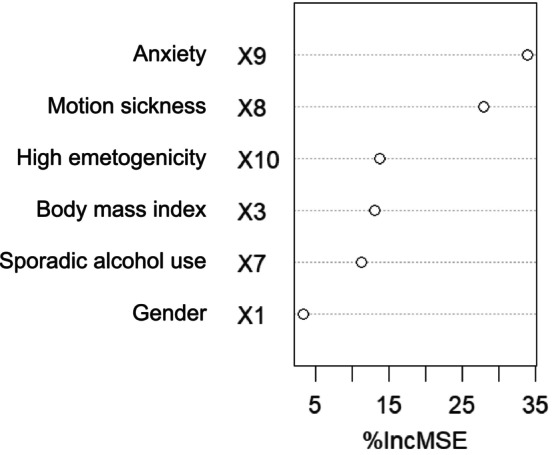
Importance ranking of risk factors for delayed nausea.

**FIGURE 2:**
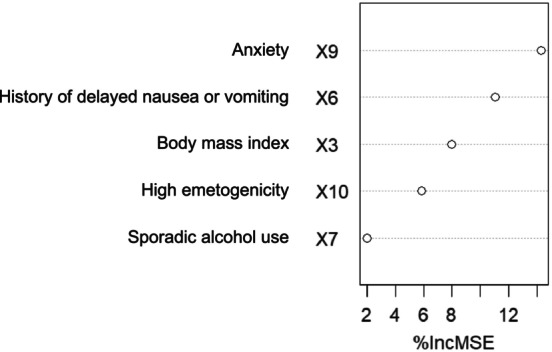
Importance ranking of risk factors for delayed vomiting.

Following the univariate analysis, the statistically significant variables were included in a random forest model to assess and rank their importance. For delayed nausea, as shown in Figure 1, the included six variables were ranked based on their importance as follows: anxiety, motion sickness, high emetogenicity, BMI, sporadic alcohol use and gender. For delayed vomiting, as shown in Figure 2, the included five variables were ranked by importance as follows: anxiety, history of delayed nausea or vomiting, BMI, high emetogenicity and sporadic alcohol use.

#### 4.3.3 Variable Filtering Through LASSO Analysis

**FIGURE 3:**
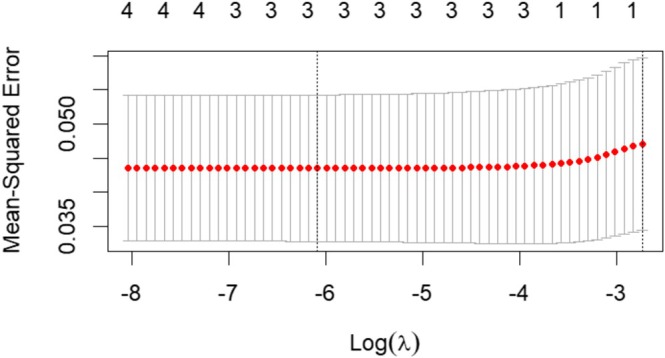
LASSO analysis of risk factors for delayed nausea.

**FIGURE 4:**
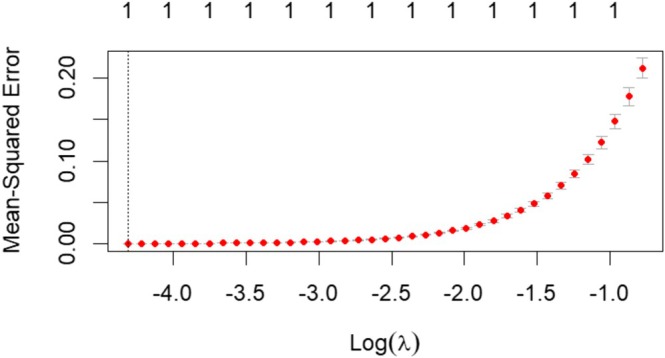
LASSO analysis of risk factors for delayed vomiting.

As shown in Figure 3, in the LASSO analysis for delayed nausea, the optimal lambda (*λ*) value was determined to be 0.00226961, where the model error was minimised. At this lambda (*λ*) value, three variables (i.e., anxiety, motion sickness and high emetogenicity) were identified as the most influential and were subsequently included in the binary logistic regression analysis. For delayed vomiting, the LASSO analysis identified that the model error was minimised at a lambda (*λ*) value of 0.01340477. At this optimal value, only one factor was selected for inclusion in the model, as illustrated in Figure 4. The single factor retained was anxiety, which was subsequently used in the binary logistic regression analysis.

#### 4.3.4 Logistic Regression Analysis

Binary logistic regression analyses were performed with delayed nausea and delayed vomiting as the dependent variables. The results, presented in Table 2, indicated that anxiety (OR = 49.337 [6.302–386.249], *p* < 0.001), motion sickness (OR = 11.978 [3.272–43.847], *p* < 0.001) and high emetogenicity of chemotherapy agents (OR = 2.792 [1.633–4.774], *p* < 0.001) were significant risk factors for delayed nausea. For delayed vomiting, anxiety (OR = 7.857 [2.774–22.256], *p* < 0.001) was shown to be the single significant risk factor.

**TABLE 2:** Multivariate analysis of risk factors for delayed CINV (*N* = 326).

Variable	*B*	SE	Wald *χ* ^2^	*p*	OR	95% CI	
Delayed nausea							
Anxiety	3.899	1.050	13.788	< 0.001**	49.337	6.302	386.249
Motion sickness	2.483	0.662	14.065	< 0.001**	11.978	3.272	43.847
High emetogenicity	1.027	0.274	14.079	< 0.001**	2.792	1.633	4.774
Delayed vomiting							
Anxiety	2.061	0.531	15.058	< 0.001**	7.857	2.774	22.256

*Note:* **p < 0.01.

Abbreviations: CI, confidence interval; OR, odds ratio; SE, standard error.

#### 4.3.5 Post Hoc Analysis

Binary logistic regression analyses were performed again using delayed nausea and delayed vomiting as dependent variables. The results showed that BMI was a significant predictor for both delayed nausea (OR = 0.880 [0.809–0.957], *p* = 0.003) and delayed vomiting (OR = 0.879 [0.786–0.984], *p* = 0.025). Furthermore, history of delayed nausea or vomiting also emerged to be an important predictor for delayed vomiting (OR = 2.907 [1.326–6.374], *p* = 0.008). The results of post hoc analysis were presented in Table 3. Given the limited number of events for delayed vomiting (*n* = 43), the post hoc multivariable model was interpreted cautiously to minimise potential overfitting.

**TABLE 3:** Multivariate analysis of risk factors for delayed CINV as post hoc analysis (*N* = 326).

Variable	*B*	SE	Wald *χ* ^2^	*p*	OR	95% CI	
Delayed nausea							
Anxiety	3.633	1.052	11.923	0.001**	37.834	4.811	297.511
Motion sickness	2.285	0.683	11.196	0.001**	9.823	2.577	37.453
High emetogenicity	1.001	0.309	10.515	0.001**	2.721	1.486	4.984
BMI	−0.128	0.043	8.929	0.003**	0.880	0.809	0.957
Gender	−0.322	0.281	1.306	0.253	0.725	0.418	1.259
Sporadic alcohol use	0.195	0.355	0.301	0.583	1.215	0.606	2.435
Delayed vomiting							
Anxiety	2.172	0.578	14.132	< 0.001**	8.773	2.828	27.221
History of delayed nausea or vomiting	1.067	0.401	7.097	0.008**	2.907	1.326	6.374
BMI	−0.129	0.057	5.050	0.025*	0.879	0.786	0.984
High emetogenicity	0.608	0.419	2.110	0.146	1.837	0.809	4.174
Sporadic alcohol use	0.130	0.523	0.061	0.804	1.138	0.409	3.170

*Note:* *p < 0.05, **p < 0.01.

Abbreviations: BMI, body mass index; CI, confidence interval; OR, odds ratio; SE, standard error.

## 5 Discussion

The present study examined the incidence and risk factors associated with delayed CINV among 326 patients undergoing chemotherapy. The overall incidence of delayed CINV was 34.7% (*n* = 113), with 32.2% (*n* = 105) experiencing delayed nausea and 13.2% (*n* = 43) experiencing delayed vomiting. Through a rigorous step‐by‐step statistical approach, including univariate analysis, random forest modelling, LASSO analysis and binary logistic regression, several significant risk factors for delayed CINV were identified, including anxiety, motion sickness and high emetogenicity of chemotherapy agents, while BMI and history of delayed nausea or vomiting emerged as important predictors that should be considered when assessing the risk of delayed CINV. These findings provide valuable insights into the multifactorial and complex nature of delayed CINV and underscore the importance of targeted interventions.

The incidence of delayed CINV in this study was lower than some previous studies, particularly those involving patients receiving highly emetogenic chemotherapy and moderately emetogenic chemotherapy. A study with 298 adult patients showed that 60% and 50% of patients receiving highly emetogenic chemotherapy experienced delayed nausea and vomiting, and 52% and 28% receiving moderately emetogenic chemotherapy had delayed nausea and vomiting (Grunberg et al. 2004). The relatively lower incidence observed in our study may be attributed to the fact that about 70% of our participants were treated with chemotherapy agents of minimal, low or moderate emetogenicity. This discrepancy underscores the critical role of chemotherapy agent emetogenicity in the development of delayed CINV, emphasising the need for a tailored approach to antiemetic prophylaxis based on the specific emetogenic potential of the treatment.

A major finding of this study was the identification of anxiety as a potent risk factor for delayed CINV. The binary logistic regression analysis revealed that anxiety significantly increased the odds of developing both delayed nausea (OR = 49.337, *p* < 0.001) and delayed vomiting (OR = 7.857, *p* < 0.001). These results are consistent with existing literature, which underscores the role of psychological factors, particularly anxiety, in exacerbating delayed CINV (Janelsins et al. 2013). Anxiety can increase the sensitivity of the vomiting centre in the brain, potentially through the hyperactivation of the hypothalamic–pituitary–adrenal axis, leading to the elevated levels of adrenocorticotropic hormone and cortisol. These hormonal changes can induce autonomic symptoms such as excessive salivation, increased heart rate, fluid retention and nausea (Di Mattei et al. 2016). This finding emphasises the critical need for comprehensive psychological assessments in patients undergoing chemotherapy.

Motion sickness emerged as another significant predictor of delayed CINV, particularly for nausea (OR = 11.978, *p* < 0.001). This finding aligns with the understanding that individuals who experience motion sickness are more prone to nausea and vomiting due to similar underlying mechanisms, including the involvement of the vestibular system (Golding 2006). The overlap in pathophysiology between motion sickness and delayed CINV suggests that patients with a history of motion sickness are particularly vulnerable to the effects of chemotherapy on the vomiting centre in the brainstem. Given these findings, it is essential for health care providers to include questions about motion sickness in their pre‐chemotherapy assessments. Patients with a history of motion sickness may benefit from tailored antiemetic strategies, including the use of vestibular suppressants in combination with standard antiemetic regimens. This proactive approach could help mitigate the risk of delayed CINV in this vulnerable population.

High emetogenicity of chemotherapy agents was also identified as a significant factor influencing the occurrence of delayed CINV, with higher emetogenicity associated with increased risk of delayed nausea (OR = 2.792, *p* < 0.001). This finding is consistent with the established role of the emetogenic potential of chemotherapeutic agents in the pathophysiology of delayed CINV, where these agents activate key neurotransmitter receptors, including serotonin (5‐HT3), dopamine (D2) and NK‐1 receptors, within the vomiting centre (Kottschade et al. 2016). Our findings underscore the need for tailored antiemetic strategies based on the emetogenicity of chemotherapy agents. The findings also highlight the significance of adhering to evidence‐based guidelines, such as the MASCC and ESMO guidelines (Herrstedt et al. 2024), which provide recommendations on antiemetic prophylaxis tailored to the emetogenic potential of various chemotherapeutic agents. Ensuring adherence to these guidelines can significantly reduce the incidence of delayed CINV, improving patient outcomes and quality of life.

Interestingly, our study identified a significant association between BMI and delayed CINV in both univariate analysis and multivariate post hoc analysis. However, this association did not persist in the LASSO analysis, suggesting that BMI may not be a robust independent predictor of delayed CINV when adjusted for other influential variables, such as anxiety and motion sickness. This finding indicated that the role of BMI in predicting delayed CINV may be more complex and dependent on other co‐occurring factors. The existing literature suggests that lower BMI could increase the risk of CINV, as malnutrition, which often accompanies lower BMI, is prevalent among patients with delayed CINV and contributes to reduced dietary intake (Davidson et al. 2012). However, clear cut‐off values for BMI that predict CINV incidence remain undefined. One study from Japan has reported that a BMI of less than 27.5 kg/m^2^ is associated with a higher risk of CINV (Kawazoe et al. 2018). But this threshold may not be applicable to our study population, where the median BMI was 23.1, a value considered normal for the Chinese population. Our findings, along with those of other studies, highlight that underweight and malnourished patients are more susceptible to frequent CINV episodes. However, further research is needed to establish precise BMI thresholds for targeted interventions, taking into account the variability in risk across different patient populations and chemotherapy regimens.

Our study also identified history of delayed CINV as an important predictor of delayed vomiting during post hoc analysis. Patients with a prior history of delayed CINV were more likely to experience recurrent episodes, likely due to a cumulative risk effect that may be related to increased sensitivity of the vomiting pathways following repeated exposure to chemotherapy (Molassiotis et al. 2016; Rapoport 2017). Previous studies have demonstrated that inadequate control of CINV during the first chemotherapy cycle can increase the risk of delayed CINV by at least sixfold in subsequent cycles, suggesting that effective early management of CINV can help mitigate its cumulative impact over time (Molassiotis et al. 2016). These findings underscore the importance of implementing aggressive prophylactic measures from the first chemotherapy cycle to reduce the risk of delayed CINV in future cycles (Rapoport 2017).

### 5.1 Strength and Limitations

This study has several strengths. The use of a robust statistical approach, including univariate analysis, random forest modelling, LASSO analysis and binary logistic regression, allowed for the identification of key risk factors and provided a comprehensive understanding of the multifactorial nature of delayed CINV. Additionally, the sample size of 326 patients in this study was sufficient to achieve meaningful statistical power, enhancing the reliability of the findings.

However, this study has several limitations. The primary limitation of this study is its cross‐sectional design, which precludes the establishment of causal relationships. The follow‐up period was restricted to three days post‐chemotherapy, which may not fully capture the extent of delayed CINV, particularly in patients who develop symptoms beyond this timeframe. Future studies applying prospective longitudinal designs will aid in gaining deeper insights into the onset and progression of CINV. Furthermore, all data originates from a large tertiary hospital in Shanghai, where clinical protocols and antiemetic regimens may be more standardised and advanced than those in primary care facilities or hospitals in economically less developed regions. This may potentially limit the generalizability of the findings to other populations and clinical settings. While the use of consecutive sampling and a diverse patient population enhanced the external validity of the results, caution should be paid when applying these findings to broader populations, as the generalisability may be constrained by similar clinical environments and patient demographics. The assessment of sleep disorders and anxiety was based on basic patient self‐reports rather than validated standardised scales, which may have compromised the precision and scientific rigour of quantifying these variables. Future research should incorporate standardised scales to more accurately elucidate the associations between sleep disorders, anxiety and CINV. The confidence interval for the impact of anxiety on CINV was quite wide. This result is unstable and should be interpreted with caution.

### 5.2 Recommendations for Future Research

Future research should focus on extending the follow‐up period beyond three days post‐chemotherapy to capture the full spectrum of delayed CINV, particularly in patients who may develop symptoms later. Multi‐centre studies are recommended to enhance the generalizability of the findings across diverse populations and health care settings. Additionally, further exploration into the relationships between BMI and history of delayed nausea or vomiting and delayed CINV is warranted, with long‐term studies to better understand the gradual impact of these factors. Finally, investigating the effectiveness of early psychological interventions, such as anxiety management, could provide valuable insights into reducing the incidence of delayed CINV, particularly among high‐risk patients.

### 5.3 Implications for Policy and Practice

The findings of this study have significant implications for both policy and clinical practice in the management of delayed CINV. The identification of anxiety, motion sickness and high emetogenicity of chemotherapy agents as key risk factors for delayed CINV underscores the need for a more comprehensive and individualised approach to CINV management. Health care policies should prioritise the integration of psychological assessments, such as routine anxiety screenings, into standard oncology care. Furthermore, the incorporation of personalised antiemetic protocols based on a patient's cumulative risk profile, including their history of motion sickness and emetogenic potential of their chemotherapy regimen, is crucial for effective prevention of delayed CINV. Clinically, this approach assists health care professionals in rapidly identifying patients at high risk of CINV prior to treatment initiation. For such high‐risk individuals, health care providers should adopt a proactive stance, initiating aggressive prophylactic measures from the first cycle of chemotherapy. These interventions include strict adherence to multi‐agent combination antiemetic regimens indicated by guidelines, the provision of enough delayed‐phase medicine reserves upon discharge and thorough patient education. This method reduces the cumulative impact of delayed CINV over multiple treatment rounds. Nursing staff should incorporate this risk assessment into their usual treatment, increasing the frequency and intensity of follow‐up for high‐risk patients. Patients and their families should be educated about the importance of preventive medication and the need to stick to a consistent dose plan. This approach can enhance patients' quality of life and treatment experience. The study highlights the importance of educating health care providers on the latest evidence‐based guidelines, ensuring that antiemetic strategies are consistently updated and tailored to individual patient needs. Implementing these practices may improve patient outcomes, enhance quality of life and reduce the overall burden of CINV on both patients and the health care system.

## 6 Conclusions

Our study examined the incidence of delayed CINV among adult patients undergoing chemotherapy, providing critical insights into the multifactorial and complex nature of delayed CINV. We identified key risk factors for delayed CINV, including anxiety, motion sickness and high emetogenicity of chemotherapy agents, along with additional important factors such as BMI and history of delayed nausea or vomiting. Our findings emphasised the need for early and aggressive prophylactic strategies, particularly from the first chemotherapy cycle, to mitigate the cumulative effects of delayed CINV. Integrating psychological support and personalised antiemetic protocols into routine oncology care could enhance patient outcomes and quality of life, underscoring the necessity for continued research and policy refinement in this area.

## Author Contributions

**Mimi Zheng:** investigation; data curation; formal analysis; investigation; software; validation; writing – original draft; writing – review and editing. **Qi Zhang:** conceptualization; methodology; writing – review and editing; project administration. **Liping Ni:** investigation; data curation; methodology. **Zheng Zheng:** methodology; project administration. **Ying Wang:** methodology; project administration; writing – review and editing. **Yiwen Chen:** investigation; data curation. **Jing Huang:** investigation; data curation. **Jian Gao:** methodology. **Quanlei Li:** conceptualization; methodology; formal analysis; project administration; writing – review and editing; **Yuxia Zhang:** conceptualization; methodology; project administration; writing – review and editing.

## Funding

Key Weak Discipline Development Fund at Zhongshan Hospital, Fudan University (XK‐082‐007).

## Ethics Statement

The study has been approved by the Medical Ethics Committee of Zhongshan Hospital, Fudan University (ethical approval reference number: B2024‐098R).

## Conflicts of Interest

The authors declare no conflicts of interest.

## Data Availability

The data that support the findings of this study are available from the corresponding author upon reasonable request.
